# Can Ponies (Equus Caballus) Distinguish Human Facial Expressions?

**DOI:** 10.3390/ani12182331

**Published:** 2022-09-07

**Authors:** Katrina Merkies, Yuliia Sudarenko, Abigail J. Hodder

**Affiliations:** 1 Department of Animal Bioscience, University of Guelph, Guelph, ON N1G 2W1, Canada; 2 Campbell Centre for the Study of Animal Reproduction, University of Guelph, Guelph, ON N1G 2W1, Canada

**Keywords:** anger, joy, sadness, laterality, experience, human discrimination

## Abstract

**Simple Summary:**

Facial expressions communicate a great deal of information that can potentially convey the affective state of the sender and facilitate approach or avoidance responses by the receiver. Reading facial expressions across species is particularly relevant for domesticated animals who rely on humans for food, shelter, safety and social relationships. In this study, 20 ponies were individually presented with facial expressions of anger, joy, sadness and neutral by two different live actors. The ponies looked with their left eye first, more often and for longer to angry expressions and with their right eye first to joy, in keeping with the theory of lateralized responses (i.e., the right brain hemisphere is activated by stimuli on the left and is predominant in emotionalized responses). The ponies distinguished between the actors, favouring one actor with shorter looking and approach times and fewer oral behaviours. Ponies with more experience as a lesson mount had lower heart rates and lower head carriage although they exhibited more oral behaviours and kept their ear on the actor more. Ponies clearly distinguish among human facial expressions but other factors also contribute to their responses to humans.

**Abstract:**

Communication within a species is essential for access to resources, alerting to dangers, group facilitation and social bonding; human facial expressions are considered to be an important factor in one’s ability to communicate with others. Evidence has shown that dogs and horses are able to distinguish positive and negative facial expressions by observing photographs of humans, however there is currently no research on how facial expressions from a live human are perceived by horses. This study investigated how ponies distinguish facial expressions presented by live actors. Trained actors (*n* = 2), using the human Facial Action Coding System, displayed four facial expressions (anger, sadness, joy and neutral) individually to twenty ponies. Heart rate and behaviors of the ponies including first monocular eye look, eye look duration (right and left side bias) and latency to approach were observed. A generalized linear mixed model (GLIMMIX) using Sidak’s multiple comparisons of least squared means determined that when exposed to anger expressions ponies looked more often with their left eye first and when exposed to joy, looked more often with their right eye first (*p* = 0.011). The ponies spent more time looking at angry expressions (*p* = 0.0003) in comparison to other expressions. There was no variation in heart rate across expressions (*p* > 0.89). Regardless of human facial expression, ponies looked longer (*p* = 0.0035), took longer to approach (*p* = 0.0297) and displayed more oral behaviours (*p* < 0.0001) with one actor than the other indicating increased arousal or negative valence. Ponies with more experience as a lesson mount had lower heart rates (*p* < 0.0001) carried their head lower (*p* < 0.0001), kept their left ear on the actor (*p* < 0.03) and exhibited more oral behaviours (*p* < 0.0001) than ponies with less experience. This study demonstrates that ponies are able to distinguish facial expressions presented by a live human, but other factors also contribute to their responses to humans.

## 1. Introduction

Facial expressions are considered an important factor in human ability to communicate with others [1]. Facial expressions allow humans to accurately assess a visual picture, associate it with a presumed underlying emotional state and determine an appropriate response; for example, the angrier looking a person is, the greater the perceived strength and thus the more motivated the receiver may be to avoid conflict [2]. This communicative ability has biological significance in terms of avoiding harmful stimuli, accessing resources, facilitating group dynamics and social bonding. Visual recognition of emotional expression is essential for processing social information and regulating interactions. For example, horses presented with photographic stimuli of conspecifics displaying negative expressions were more likely to avoid those photographs than when exposed to positive expressions, which they were more likely to approach [3].

Domestication has led to a reliance of animals on humans for provision of food, shelter, safety and social interactions in a mutualistic relationship [4]. Thus, it would be advantageous for non-human animals to be able to interpret human facial expressions for facilitation of this relationship. The same underlying mechanisms for facial expressions appear to be conserved across species [5]. Goats allowed to interact with photos of a happy or angry human spent more time with the happy face [6]. “Social eavesdropping” on positive and negative human-horse interaction video scenarios resulted in horses responding with more positive behaviours when watching the positive scenario and vice versa [7]. Horses presented with photographs of humans displaying angry or happy expressions followed by meeting the live human presenting a neutral expression several hours later displayed more arousal behaviour toward the neutral person associated with the previously viewed angry face [8]. Thus, recognition and remembrance of human emotions allows an animal to adjust their behaviour accordingly.

Human faces express emotion asymmetrically with stronger expression exhibited on the left side of the face [9]. This is likely due to brain lateralization whereby stimuli processed in right brain hemisphere activate the left side of the body and vice versa [10]. The left-brain hemisphere is associated with social and learned behaviour requiring decision-making and patterns of response while the right brain hemisphere is emotionally dominant, associated with emotional responses to novel, unpredicted and potentially dangerous situations [9,10,11]. In non-human animals, research on brain laterality has focused on the connection between the hemispheres and eye gaze, where left brain hemisphere processing is attributed to a right eye gaze bias and the right brain hemisphere processing is attributed to a left eye gaze bias [10]. Individuals with a more strongly lateralized response also have quicker response times, potentially linking laterality and emotional processing with cognition [10,11,12]. Dogs viewing negative human facial expressions such as anger displayed a left eye gaze bias in comparison to positive human facial expressions such as joy [13]. Horses are considered a good subject to observe the effects of brain hemispheric activity on eye gaze due to the lateral placement of the eyes [14]. Horses possess only a narrow field of binocular vision directly in front of them and rely mainly on monocular vision to monitor their surroundings [15]. This makes the eye the horse is observing external visual stimuli with easily identifiable. Horses confronted with photographs of humans displaying positive and negative facial expressions exhibited an increased look duration with the left eye when observing anger while simultaneously experiencing an increase in heart rate [16]. These results show a clear relationship between a left side bias towards a negative visual stimulus influencing the physiological state of the animal, suggesting that horses view stimuli that they perceive to be negative with their left eye.

Understanding how horses perceive emotional information from humans can lead to a greater understanding of their affective state and cognitive abilities [5], enabling effective approaches to daily care and training, ultimately improving the horse-human bond [17]. Although research has provided evidence of horses distinguishing human facial expressions through the use of photographs, there is no research on their initial response to live humans. This study aimed to investigate how ponies perceive human facial expressions when exposed to live humans. It was hypothesized that ponies exposed to negative facial expressions (anger and sadness) would have an increased look duration with their left eye, increased arousal behaviours and increased heart rate and latency to approach the human in comparison to the other expressions (joy and neutral).

## 2. Materials and Methods

### 2.1. Subjects

In this study, 20 ponies (Equus caballus) of mixed breed and sex (10 geldings, 10 mares) participated in the study. Their ages ranged from 6–25 years old (average 10.5 ± 5.9 years). Ponies were housed in groups on pasture and fed free choice grass hay. All ponies had prior experience under saddle; however, the level of experience with each pony varied between little experience (green; *n* = 8) and highly experienced and regularly used in the lesson program (school; *n* = 12; Table 1). All ponies were accustomed to being handled by different people.

Two human actors with similar appearance were selected for the trials to ensure little variation between subjects: two Caucasian females of similar height (average 155 cm) and weight (average 54 kg) with long brown wavy hair.

### 2.2. Testing Area

**Figure 1:**
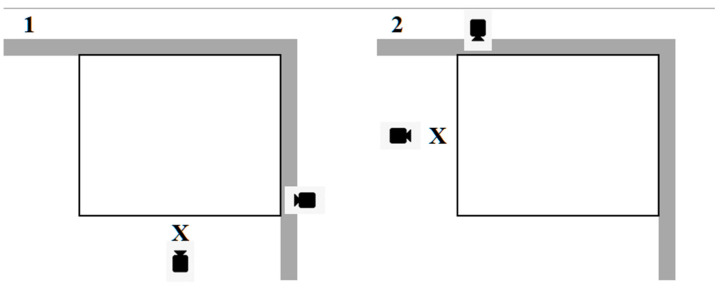
The testing pen where observations of ponies (*n* = 20) responding to differing human facial expressions were conducted with two different orientation set-ups. The pen was 5 m^2^ constructed using two walls of the indoor arena and two sides made of barriers 0.5 m high. The actor (X) portraying the facial expression was seated on a chair placed 1.0 m from the edge of the pen. Two video cameras were placed to capture the behavioural responses of the ponies in two different orientations: one camera was always behind the actor and second camera was on the perimeter of the pen either to the right (**1**) or to the left (**2**) of the actor.

A 5 × 5 m square enclosure inside an indoor arena familiar to the ponies was constructed in one corner of the arena using 0.5 m high barriers on the two open sides. The actor was seated 1.0 m outside of the pen at the centre of one barrier wall. To control for side biases, two orientations were used in semi-randomized order such that each pony was tested in each configuration for equal numbers of trials (Figure 1). Video cameras were located on the perimeter of the testing area. One camera (Panasonic HC-X900M), mounted on a tripod, was placed 1.6 m from the edge of the pen directly behind and above the actor’s head to focus on the front of the face and body of the pony as they interacted with the human. The second camera (Sony Handycam HDR-CX405) was placed at a height of 1.0 m and a distance of 2.5 m directly to the right of the actor in orientation 1 and to the left of the actor in orientation 2 to focus on the lateral side of the face and body of the pony. Prior to data collection, the ponies were subjected to a habituation period in the testing pen as described below.

### 2.3. Training and Habituation

Prior to testing, ponies underwent training to associate a clicker device with positive reinforcement (i.e., a carrot). The purpose of the training was to habituate each pony to the pen and to encourage the pony to actively seek interaction with the actor during the testing. Initial training was carried out one week prior to testing. One researcher (KM), standing beside the pony loose in the pen, would “click” and immediately reward the pony with a treat (carrot). This was repeated six times to ensure the pony associated the “click” with positive reinforcement. The researcher then created a 2 m distance from the pony, “clicked” and rewarded the pony’s approach with a carrot. This was repeated until the pony immediately approached the researcher after the “click”. Finally, the researcher stood outside the pen and proceeded to “click” followed by a food reward when the pony approached. All ponies successfully associated the clicker with a food reward. A second training session was repeated three days prior to testing to refresh the ponies and ensure training was complete.

### 2.4. Facial Expressions

Prior to testing, both actors underwent training in facial expression display. The Facial Action Coding System (FACS [18]) was utilized to portray the human facial expressions of interest due to its objectivity [19] and prior use examining horse response to human facial expressions [16,20]. One researcher (AJH) delivered the training sessions to the two actors. Training included an instructional manual describing facial action units (AU) in accordance with specific muscle groups to use with each facial expression. Four facial expressions were tested using FACS (Table 2):

1. Neutral: All muscles are relaxed
2. Anger: AU 4 + 5 + 7 + 23
3. Joy: AU 6 +12, teeth are to be shown
4. Sadness: AU 1 + 4 + 15

In the training session the researcher, while using visual imaging provided by FACS, displayed each of the expressions indicating the muscles used for each and the actors would mimic until they felt confident in displaying the expression. A validation test required each actor to approach four random human subjects individually and display each of the four facial expressions in random order in eight repetitions, resulting in 128 displays. The subjects were queried on which expression they believed was being presented. Both actors successfully completed the training with all subjects scoring 80% and above for correct identification of the facial expression displayed.

### 2.5. Data Collection

Ponies were equipped with heart rate monitors (Polar RS800, Lachine, QC) at least 15 min before entering the testing pen and for the duration of each test.

Each pony was individually exposed to each of four facial expressions in randomized order from each of the two actors. Actors both wore a short-sleeve grey shirt and black pants with hair tied back and no facial jewelry present. Actors presented the same posture: sitting in a chair 1.0 m outside the perimeter of the pen with shoulders back, hands resting in lap and feet together. The face of each actor when seated was 120 cm from the ground. Both actors were unfamiliar to all ponies.

A handler brought each pony individually to the testing pen and held them facing the actor at a distance of 1.0 m. At a signal from the researcher who was standing outside of the pen near the side video camera and out of sight of the pony, the handler released the pony and stepped to the rear of the pen to sit in a chair facing away from the pony and the actor. Simultaneously the actor used the clicker and displayed the facial expression indicated by the researcher in a randomized order continuously for 60 s. After this time, the trial stopped and was repeated for the remaining three facial expressions. Once all expressions were complete, the pony was removed from the pen and another pony was introduced in a randomized order until all twenty ponies observed the actor. This was repeated for the second actor on a separate day.

### 2.6. Behavioural Observations

All tests were video recorded for retrospective analysis of relevant behaviours (Table 3). Videos were uploaded and behaviours were coded using the Behavioural Observation Research Interactive Software (BORIS version 7.6, Torino, Italy [21]). To reduce observer subjectivity, two observers blind to the treatment viewed all of the videos, with each observer coding only specific behaviours. Eye behaviours including number of bouts and duration of left, right and binocular gaze at the actor were recorded as frequencies and durations, respectively. First monocular look indicated which single eye the pony used to first look at the human (i.e., left or right eye). Latency to approach recorded the time the pony took to approach the actor; if the pony never approached latency was scored as 60 s. Head height, ear orientation and distance of the pony from the actor were noted in 1 s intervals for each 60 s test. Head height was coded as high head height = 1, even head height = 2, low head height = 3. Ear orientation was coded by observing only the pony’s left ear where 1 = left ear on human and 2 = left ear not on human. Distance from the actor was coded as 1 = less than 1 m away, 2 = 1–2 m away and 3 = more than 2 m away. Oral behaviours counted the total number of behaviours performed over 60 s.

### 2.7. Data Curation and Statistical Analysis

To determine left and right eye look duration, a laterality index (LI) was used according to [16]: LI = (L − R)/(L + R + B) × 100, where L = total left eye look duration, R = total right eye look duration and B = total binocular look duration for each 60 s trial. If the resulting number was positive the pony exhibited a left eye gaze bias and if the number was negative, then the pony exhibited a right eye gaze bias.

Statistical analyses were carried out using SAS (version 9.4, Toronto, ON, Canada) where *p* values < 0.05 were considered significant. First monocular look was analyzed using a binary distribution (left or right eye) and a frequency analysis determined the total number of first monocular looks for each eye for each facial expression. Analysis for total look duration (L + R + B) determined any difference in the total time the ponies spent observing the actors across expressions. Eye look bouts, laterality index, latency and oral behaviours were analyzed as frequency data, while heart rate, distance from actor, head height and ear orientation data were analyzed separately per second using a log transformation as they did not show a normal distribution (Kolmogorov-Smirnov, *p* < 0.001).

Baseline HR was calculated from HR recordings collected during the 5 min prior to the start of each trial. A Pearson correlation coefficient was calculated and revealed no differences between baseline HR and HR during the trials (*r* (158) = 0.74, *p* < 0.0001). Additionally, heart rate did not differ over the 60 s of each trial. Thus, an average heart rate collected during each trial was calculated in beats per minute (bpm) for analyses.

A General Linear Mixed Model (GLIMMIX) procedure with repeated measures determined relationships between observed behaviours and heart rate with facial expression, actor, pony experience and their interactions. Orientation of the test pen was included as a random effect. Sidak’s post hoc tests determined differences among multiple comparisons of least squared means.

## 3. Results

### 3.1. Effect of Human Facial Expression

#### 3.1.1. First Monocular Look

**Figure 2:**
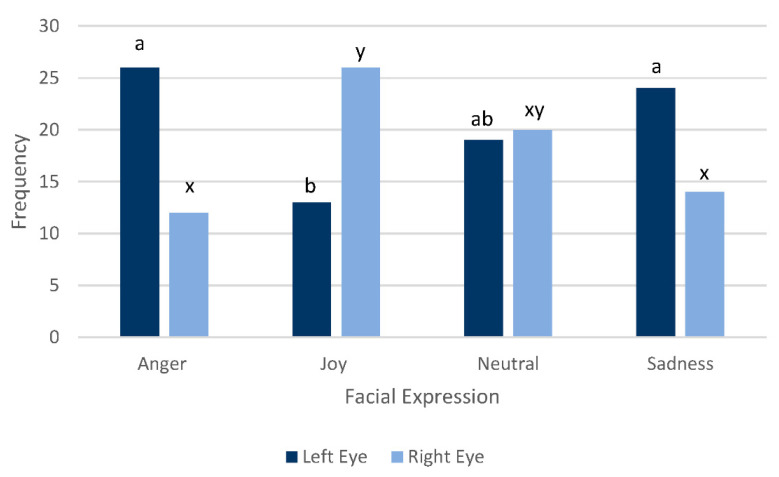
The number of first monocular looks with either the left or right eye of ponies (*n* = 20) looking at actors portraying facial expressions of anger, joy, neutral and sadness over a 60 s observation period. a, b and x, y differ across facial expressions *p* = 0.011.

There was a significant difference in the single eye used to first look at the actor across the four facial expressions (F(3129) = 3.86, *p* = 0.011). Ponies looked more often with their left eye first when viewing angry and sad facial expressions compared to joy. Ponies looked more often with their right eye first when viewing the joy expression compared to anger and sadness. Neutral facial expressions resulted in an almost even distribution of left and right eye first monocular looks (Figure 2). There was no significant difference between first monocular look and actor (F(1129) = 0.07; *p* = 0.7930) or pony experience (F(1129) = 1.81; *p* = 0.1811). There were six instances (out of 160 trials) where the pony did not turn their head at least 45° to look at the actor at all.

#### 3.1.2. Laterality Index

**Figure 3:**
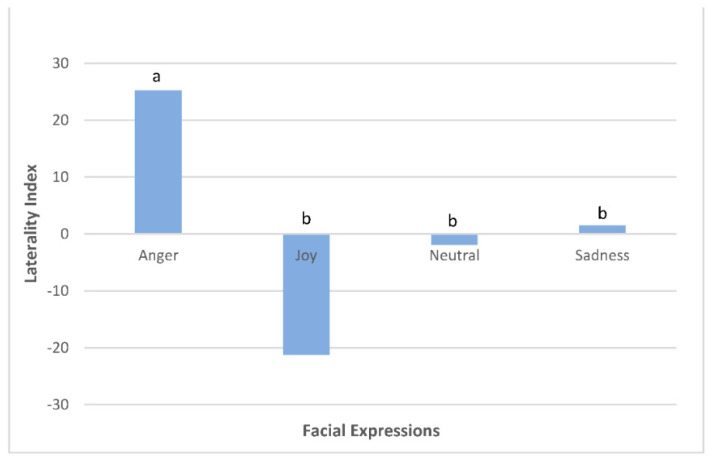
Laterality index used to calculate the duration of time ponies (*n* = 20) spent looking with their left eye (positive number) or right eye (negative number) when looking at actors portraying facial expressions of anger, joy, neutral and sadness over a 60 s observation period. a, b differ across facial expressions *p* = 0.0003.

The look duration for each eye was calculated by a laterality index (LI = (L − R)/(L + R + B) × 100 where L, R and B refer to left, right and binocular look duration, respectively), with positive numbers indicating a left eye gaze bias and negative numbers indicating a right eye gaze bias. Ponies spent more time looking with their left eye when exposed to anger in comparison to joy, neutral and sadness facial expressions (F(3137) = 6.82, *p* = 0.0003; Figure 3). There was no effect of actor (F(1137) = 0.15, *p* = 0.7036) or pony experience (F(1137) = 0.05, *p* = 0.82) on laterality index.

#### 3.1.3. Eye Look Bouts

Ponies switched between left, right and binocular eye looks at the actor multiple times throughout each 60 s trial. Ponies exhibited more bouts of left monocular eye looks toward angry (1.7) facial expressions compared to joy (0.85), sadness (1.33) or neutral (1.13) (F(3136) = 5.18, *p* = 0.002). Ponies tended to more bouts of right monocular eye looks when viewing joy (1.53) facial expressions compared to anger (1.0) (F(3136) = 2.30, *p* = 0.0803). There was no difference in the number of bouts of binocular eye looks regardless of facial expression (F(3136) = 0.65, *p* = 0.5828).

#### 3.1.4. Heart Rate

There was no effect of facial expressions on pony heart rate (F(3137) = 0.20, *p* = 0.8977). The average heart rates for ponies exposed to the facial expressions were 45.0 ± 16.26 bpm for anger, 44.4 ± 15.22 bpm for joy, 45.4 ±13.82 bpm for neutral, and 45.2 ±15.31 bpm for sadness.

#### 3.1.5. Behaviour

There was a significant difference in the average frequency of oral behaviours displayed by the ponies when presented with the four different facial expressions (F(3137) = 6.86, *p* = 0.0002). Oral behaviours occurred more often with neutral (4.0 ± 7.67/min) compared to anger (2.9 ± 5.13/min), joy (2.8 ± 5.62/min) and sadness (2.5 ± 3.90/min) facial expressions.

Ear orientation of the ponies differed based on facial expression (F(39,556) = 9.16, *p* < 0.001); ponies focused their left ear on the actor more often with joy and sadness expressions compared to anger. Ponies stood farther away from the actor when they portrayed joy and sadness expressions compared to anger and neutral (F(39,556) = 10.50, *p* < 0.0001). There was no effect of facial expressions on the ponies’ head height (F(39,556) = 1.95, *p* = 0.1199).

### 3.2. Effect of Human Actor

**Figure 4:**
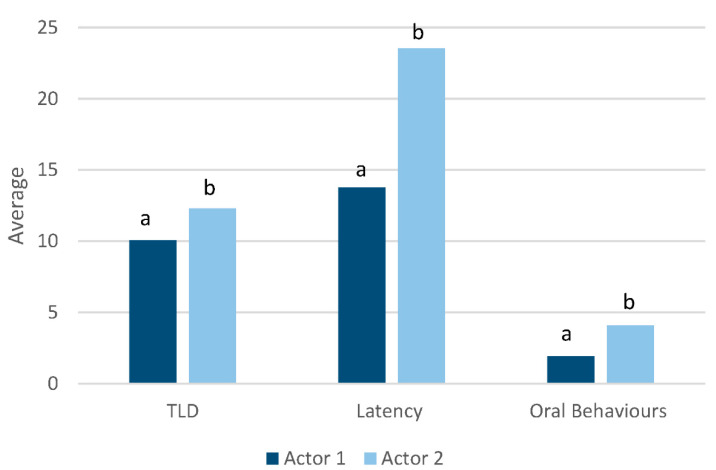
Observations of total look duration (TLD; s), latency to approach (s) and oral behaviours (frequency/min) displayed by ponies (*n* = 20) when interacting with two different actors portraying facial expressions of anger, joy, neutral and sadness over a 60 s observation period. a, b differ between actors *p* < 0.03.

Average total look duration (TLD = left eye + right eye + binocular duration) over the 60 s trials did not differ when the ponies were presented with differing facial expressions (F(3142) = 0.98, *p* = 0.4019), however there was an effect of actor on average total look duration (F(1142) = 8.80, *p* = 0.0035; Figure 4); ponies spent more time observing Actor 2 (12.3 ± 8.50 s) in comparison to Actor 1 (10.1 ± 7.43 s).

Although latency to approach the actor did not differ according to the facial expressions presented (F(3143) = 0.65; *p* = 0.5825), there was a significant difference between the actors (F(1137) = 4.83; *p* = 0.0297; Figure 4); ponies took longer to approach Actor 2 (23.5 ± 23.57 s) in comparison to Actor 1 (13.8 ± 18.57 s).

While there was no interaction between actor and facial expression on the average frequency of oral behaviours displayed by the ponies (F(3143) = 1.44; *p* = 0.2329), there was a significant difference between the two different actors (F(1137) = 60.38, *p* < 0.0001; Figure 4). Oral behaviours occurred more often with Actor 2 (4.1 ± 7.39/min) in comparison to Actor 1 (2.0 ± 2.96/min). Heart rates of ponies did not differ toward the actors (F(1137) = 0.47, *p* = 0.4946).

### 3.3. Effect of Pony Experience

There was no interaction between pony experience and their latency to approach the actor displaying different facial expressions (F(3143) = 0.04; *p* = 0.9903). There was a significant difference between heart rate and pony experience (F(1137) = 96.3, *p* < 0.0001). Ponies with less experience (green) had a higher heart rate (52.8 ± 18.84 bpm) in comparison to ponies with more experience (school) who had a lower average heart rate (39.8 ± 8.62 bpm).

Ponies with more experience (school) had a higher frequency of oral behaviours (4.33 ± 6.84 occurrences/min (F(1137) = 56.21, *p* < 0.0001) in comparison to ponies with less experience (green) (1.05 ± 2.32 occurrences/min). Ponies with more experience oriented their left ear on the actor more often (F(1143) = 4.61, *p* < 0.03) and spent more time with their head lowered (F(1153) = 40.42, *p* < 0.0001) when interacting with the actors in comparison to ponies with less experience.

## 4. Discussion

In the current study, ponies distinguished among human facial expressions by exhibiting a left eye gaze bias (first monocular look) with longer duration over more bouts toward angry faces, a right eye gaze bias toward joy faces and keeping their left ear on the actor while standing farther away from sadness and joy faces. Ponies responded differently to the individual actors, looking longer, taking longer to approach and performing more oral behaviours with one actor compared to the other. Ponies with more experience as a lesson mount had lower heart rates, kept their ear on the actor more, performed more oral behaviours and held their head lower compared to greener ponies.

Expressions of emotion, whether by facial expressions or physiological or biochemical responses, are highly similar across diverse species [5] thus they are believed to be innate action patterns with universal brain activation. However, emotion is more than simple reflex, requiring cognitive assembly of experience, current physiological state and context [26]. This allows for emotional responses to be generalized and recognized both within and across species. Expression of emotion communicates critical information. Insight into another’s emotional state has particular relevance for determining an individual’s reaction in light of resource acquisition or avoidance and survival, and this function may be highly adaptive for social species such as horses [3,16].

As human relationships with horses continue to evolve into mutual partnerships and “oneness” [27] communication across these two species becomes increasingly important. Efforts to understand equine facial expressions have resulted in a facial action coding system (FACS) specific for horses [28] that can aid in recognizing their underlying affective states. Equally important is to examine how horses view and understand humans. Given the similarity in muscle activation for different facial expressions across species [5,28] it is not surprising that horses can recognize and react to human facial expressions. The results presented here support the findings of others, namely that horses look with the left eye first and longer toward angry faces [8,16] however this is the first report of discrimination of facial expressions in live humans. Multimodal studies showed horses increased approach behaviour to a novel object when humans used encouraging facial expressions paired with relaxed body posture and positive vocal expressions, and increased vigilance behaviour when humans displayed an anxious facial expression with tense body posture and negative vocal expressions [29]. Similarly, horses followed human gaze less often when paired with a disgusted face and negative verbiage [30]. While multiple sources of information from a human may support a horse’s response to a scenario, it appears that human facial expression alone is enough to determine a response.

Ponies stood farther away from the actor when viewing joy and sadness facial expressions. This was an unexpected result as it was anticipated that ponies would be more avoidant (stand farther away) of angry expressions. However, it could be that the angry facial expression elicited more investigative behaviour from the ponies since they also looked at the actor with the angry expression more often. Others have postulated that positive emotions (happiness) are more difficult to discriminate or are less of an immediate threat [30], thus do not require immediate action on the part of the observer. Ponies in the current study kept their left ear on the actor displaying sadness and joy more often than with the other facial expressions. Ear position is believed to indicate the direction of focused visual attention [31] meaning left ear focused on the actor would equate to left eye look. This holds with lateralized responses toward sadness facial expressions but is perplexing in regards to the joy expression. However, ponies are able to move each ear independently and these results only focused on left ear orientation, perhaps missing important information supplied by the right ear. It could be also that the ponies were evaluating the actor before deciding on their response particularly since they had been primed to expect a food reward upon hearing a click, but the food reward did not appear during the trials. This potentially created an expectancy violation which Nakamura et al. [20] showed resulted in longer attention. Additionally, the actors remained still during their interactions with the ponies and did not follow through with any physical movement that may ordinarily accompany sadness and joy facial expressions. Similar to the results from Smith and colleagues [16] ponies in the current study did not show any change in heart rate regardless of facial expression, actor or pony experience. This would indicate that the scenario was not considered stressful enough to warrant any physiological changes.

Emotional responses of animals to various situations and environments are an area of interest to behavioural researchers. While physiological changes in heart rate or cortisol can inform us of arousal states, they may not identify the valence of the associated emotion (i.e., heart rate can increase in response to both positive and negative stimuli [14]). For this, behavioural responses add a great deal of information to assist in characterizing concurrent emotions [32]. In particular, laterality has received much attention with a general acceptance of decussated innervations: that is, activation of the right brain hemisphere is reflected in left motor or sensory responses, and left-brain hemisphere activation is reflected in right motor or sensory responses [9]. Furthermore, the right hemisphere is attributed to processing of emotions and negative stimuli while the left hemisphere is related to social and learned behaviour [9,10,11]. Investigation into laterality as an expression of emotional valence determined that horses tend to look at objects associated with a negative experience with their left eye [33]. Furthermore, horses with a higher emotionality index also looked at a novel object preferentially with their left eye [34]. In reports involving the response of horses to human facial expressions, invariably they viewed the angry faces with their left eye [3,8,16]. Ponies in the current study presented with a joy facial expression exhibited a first monocular look at the actor and spent more time looking with their right eye, which contrasts the evidence reported by Smith and colleagues [16] who did not find a first monocular right eye look in horses viewing happy facial expressions. This could be due to subtle differences in the portrayal of happy versus joy expressions even though they utilize the same action units, or could be due to different intensities of response to a live actor versus a photograph. Dogs find it harder to recognize individual people from a photo than a live person [35]. Additionally, the results from Smith et al. [16] have been called into question. Schmoll [36] undertook an arguably more correct statistical approach using Smith et al.’s [16] data set and found no effect of human facial expression on first monocular look or laterality index. Schmoll [36] reported an overall left eye gaze bias regardless of facial expression and attributed this to the influence of the experimenter always standing on the horse’s left side (albeit facing backward) in Smith et al.’s [16] study. The current study used the same statistical approach as did Schmoll [36] and the handler effect was eliminated by allowing the pony to roam free within the pen.

While the results presented here show ponies exhibiting a unique response to differing human facial expressions, other aspects of the ponies’ responses did not appear to rely on facial expression information. Behaviours such as head position, lip licking and vacuum chewing were gathered as higher head position [23,24,32] and increased oral behaviours [25,32,37] have been correlated to increased arousal or negative valence. Compared to ponies who had little experience as a lesson mount, ponies who were regularly used in a lesson program had lower heart rates and held their head lower, suggestive of lower arousal or positive valence, but contrarily kept their ear on the actor more and performed more oral behaviours suggestive of higher arousal or negative valence. Experience works to decrease arousal responses through habituation—a decrease of behavioural responses through repeated stimulation [38]. Baragli and colleagues [39] evaluated behaviours of lesson horses exposed to a novel object test and found that older horses were less responsive than younger horses. Similarly, Wathan et al. [3] found that younger horses looked longer toward photos of conspecifics regardless of the facial expression of that horse. The older ponies in the current study were likely less responsive due to experience, but that experience may also contribute to higher vigilance of the human in case they were required to do some activity [32].

Individual human characteristics also contribute to horses’ processing of various stimuli. While an attempt was made to utilize two actors with similar physical appearance and the ponies were trained prior to data collection to approach humans using positive reinforcement to create a positive perception [40], the ponies nevertheless responded by consistently favouring one actor, approaching quicker and displaying fewer oral behaviours than with the other actor. Others have shown that even when presented with identical twins, horses were able to distinguish between them [41]. The actor more favoured by the ponies in the current results also happened to have more experience with equids in general. Previous research has shown that horses approached humans experienced with horses quicker than those who were not [42].

Actors in this study were not professionally trained to portray the various facial expressions and the facial expressions were merely depicted, not indicative of an underlying affective state. Thus, the visual display of the expression may not have reflected a felt emotion [43]. However previous research showing horses’ ability to discriminate human facial expressions were based on photographs [8,16] which by their nature do not convey felt emotions. A common practice used in human psychology is to induce an emotional state that is accurately reflected by the facial expression [44]. Further research in this area could observe how horses respond to humans in genuine joyous or angry scenarios.

## 5. Conclusions

This study provides the first evidence that ponies can distinguish among human facial expressions presented by live humans. Angry faces resulted in activation of the right hemisphere as ponies viewed the human first, more often and longer with their left eye. However, ponies also responded differently to the individual human actors regardless of facial expression, and experience with humans also played a role. While human facial expression can affect ponies’ response to humans, they also appear to evaluate other aspects of human presentation. Continued research will help to understand what human factors are most salient and how their own internal affective state may be influenced.

## Figures and Tables

**Table 1:** Characteristics of ponies (*n* = 20) used in the research trials examining their response to human facial expressions.

Pony	Age (Years)	Sex *	Experience Level ^Ŧ^
Allan	6	G	School
Alli	8	M	School
Avi	6	M	Green
Bailey	16	M	School
Cassidy	25	M	School
Chino	6	M	Green
Guppy	19	G	School
Jamaica	6	G	Green
Java	6	M	School
Kermit	6	G	Green
Lily	16	M	School
Mel	15	G	School
Prada	12	M	School
Peewee	12	G	School
Rex	6	G	Green
Sterling	20	M	School
Tori	6	M	School
Tonto	6	G	Green
Turtle	7	G	Green
Volt	7	G	Green

* G = gelding; M = mare. ^Ŧ^ school = highly experience; green = inexperienced).

**Table 2:** Description of facial action units (AU) used by human actors displaying different facial expressions to ponies (*n* = 20). Information taken from FACS with the corresponding facial muscles and example visual images for expressions of anger, joy, sadness and neutral [18].

AU in FACS	Description	Facial Muscle	Visual Imaging
1	Inner Brow Raiser	*Frontalis, pars medialis*	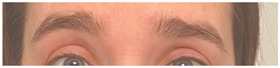
4	Brow Lowerer	*Depressor Glabellae, Depressor Supercilli, Currugator*	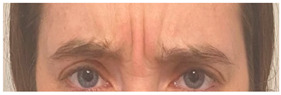
5	Upper Lid Raiser	*Levator palpebrae superioris*	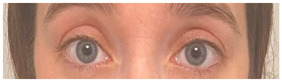
6	Cheek Raiser	*Orbicularis oculi, pars orbitalis*	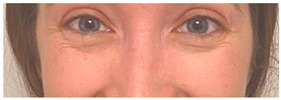
7	Lid Tightener	*Orbicularis oculi, pars palpebralis*	* 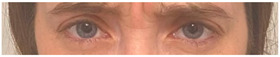 *
12	Lip Corner Puller	*Zygomatic Major*	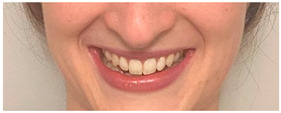
15	Lip Corner Depressor	*Depressor anguli oris (Triangularis)*	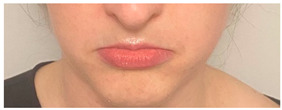
23	Lip Tightener	*Orbicularis oris*	* 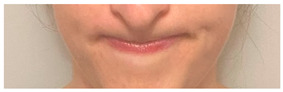 *

**Table 3:** List of behaviours recorded when ponies (*n* = 20) were exposed to human actors portraying various facial expressions for 60 s. Adapted from [22,23,24,25].

Behaviour	Description
First monocular look	The single eye that the pony first used to look at the human (right or left). The pony must turn their head at least 45° (from the central axis of the face) to achieve this.
Left eye look	Time spent with pony’s head turned at least 45° to the left (from the central axis of the face) and using their left eye to look in the direction of the human.
Right eye look	Time spent with pony’s head turned at least 45° to the right (from the central axis of the face) and using their right eye to look in the direction of the human.
Binocular eye look	Time spent with the pony’s head directly facing the human (from the central axis of the face).
Latency to approach	Time taken for the pony to move in the direction of the human (more than one step with any one foot). The pony did not have to reach or touch the actor.
Head—Above withers	Head and neck elevated with the muzzle above the chest.
Head—Even with withers	Head and neck even (muzzle at chest level).
Head—Below withers	Head and neck lowered with the muzzle below the chest.
Ear orientation	The direction and position of the left ear relative to the actor—either toward actor or away from actor.
Distance	The distance in m of the pony’s front feet from the actor.
Oral behaviours	Included lip licking (tongue extending beyond oral cavity) and chewing.

## Data Availability

Raw data can be accessed at https://data.mendeley.com/datasets/p2ywt67xmr/1.
